# Night and shift work patterns and incidence of type 2 diabetes and hypertension in a prospective cohort study of healthcare employees

**DOI:** 10.5271/sjweh.4104

**Published:** 2023-09-01

**Authors:** Andreas Viklund, Tomas Andersson, Jenny Selander, Manzur Kader, Maria Albin, Theo Bodin, Mikko Härmä, Petter Ljungman, Carolina Bigert

**Affiliations:** 1 Institute of Environmental Medicine, Karolinska Institutet, Stockholm, Sweden.; 2 Centre for Occupational and Environmental Medicine, Region Stockholm, Stockholm, Sweden.; 3 Work Ability and Working Careers, Finnish Institute of Occupational Health, Helsinki, Finland.; 4 Department of Cardiology, Danderyd University Hospital, Sweden.

**Keywords:** consecutive night, healthcare worker, metabolic disorder, night shift, night work, occupational exposure, occupational health, register data, Stockholm, Sweden, work schedule

## Abstract

**Objectives:**

This study aimed to evaluate effects of night and shift work patterns on type 2 diabetes (T2D) and hypertension in a longitudinal study, with detailed information on working hours.

**Methods:**

The cohort comprised about 28 000 nurses and nursing assistants employed for more than one year 2008–2016 in Stockholm, Sweden. The employee register held detailed individual information on daily working hours. Information on diagnoses came from national and regional registers. Hazard ratios (HR) and confidence intervals (CI) were estimated by discrete-time proportional hazard models, adjusting for sex, age, country of birth, and profession.

**Results:**

During follow-up in 2013–2017, we identified 232 cases of T2D and 875 of hypertension. We observed an increased risk of T2D, but not hypertension, among employees who worked only night shifts the previous year (HR 1.59, 95% CI 1.02–2.43) and those with intensive shift work (>120 afternoon and/or night shifts the previous year: HR 1.67, 95% CI 1.11–2.48) compared to only day work. There was a non-significantly increased risk of T2D related to mixed day and afternoon shifts (HR 1.34, 95% CI 0.97–1.88). We observed tendencies in increased risk of T2D related to frequent spells of ≥3 consecutive night shifts and with number of years with exclusive (but not mixed) night work.

**Conclusions:**

Permanent night work and frequent afternoon and/or night shifts were associated with an increased risk of T2D the following year, but not hypertension. The T2D risk was, to some extent, affected by frequent spells of several night shifts in a row and by cumulative years with permanent night work.

It is estimated that about 21% of the working population in Europe and about 40% of healthcare employees are engaged in shift work with or without night shifts (1). Working time patterns like night and shift work have been suggested to increase the risk of chronic health issues like hypertension (2, 3) and metabolic disorders, especially type 2 diabetes (T2D) (4–8). An understanding of what types of work patterns are the most associated with increased risk of such diseases would facilitate developing less harmful work schedules for those who need to work shift and night. There is much to be gained as hypertension is regarded as the leading cause of cardiovascular disease worldwide (9) and T2D, also a risk factor for cardiovascular disease, as one of the leading causes of human suffering and deaths worldwide (10). The association between night and shift work and cardiovascular disease, in which hypertension and diabetes may act as mediators, has been summarized in several reviews (11–14).

Potential mechanisms linking night and shift work to diabetes and hypertension include physiological mechanisms such as disruption of the circadian rhythm, hormonal changes and physiological stress mechanisms, as well as behavioral factors (8, 15).

Although there is accumulating evidence that night and shift work may affect the risk of diabetes and hypertension, previous studies report varying findings. Also, many studies have limitations in form of imprecise exposure data, with self-reported information on working hours and schedules, or lack of a longitudinal approach.

In this longitudinal study, we have register-based exposure data with detailed information on working hours for each employed individual in a cohort of Swedish healthcare employees. The aim was to evaluate the effects of different night and shift work patterns on the risk of incident T2D and hypertension.

## Methods

### Study design and population

This is a prospective cohort study including healthcare employees who were employed for over a year from 1 January 2008 to 31 December 2016, in the Region Stockholm, Sweden. More details about the formation of the cohort and the study design have been described previously (16). In summary, the employees were identified from a computerized administration employee register (HEROMA). We included occupational groups with a high proportion of night work, namely nurses and nursing assistants including caregivers, personal assistants and accommodation assistants. Physicians were not included, due to insufficient information on night work and working hours. By record linking to the Population Register at Statistics Sweden we got information on vital status and country of birth.

From the total cohort of nurses and nursing assistants (N=29 541) we completed an analytical subsample to investigate the risk of T2D. We excluded employees with a previous diagnosis of any type of diabetes and employees who had previously been prescribed any medication for diabetes [Anatomical Therapeutic Chemical (ATC) group: A1 – insulin and oral glucose-lowering drugs] before the first employment day or within the first 12 months of employment, as well as those who were not under risk anytime between 2013 and 2017. The patient registers dated back to 1998 for inpatient care and 2001 for outpatient care. The National Prescribed Drug Register (at the National Board of Health and Welfare) dated back to 2005.

Similarly, we completed an analytical subsample to investigate the risk of hypertension by excluding employees with a previous diagnosis of hypertension and employees who had previously been prescribed any type of medication that may be used in treatment of hypertension (ATC groups: C02, C03, C07, C08 and C09). The final samples comprised 28 481 employees (25 065 women and 3 416 men) in the T2D cohort and 23 280 employees (20 374 women and 2906 men) in the hypertension cohort. The inclusion and exclusion procedure for the cohorts is presented in a flowchart (supplementary material, URL, figure S1).

### Exposure assessment and classification

The information on working hours was obtained from Region Stockholm´s employee register HEROMA. Detailed individual information on day-by-day working hours was available from 1 January 2008 to 31 December 2016 in addition to information on occupation.

Three types of shifts were identified: day, afternoon, and night shifts (see definitions in table 2). For each year, the cohort participants were additionally classified within either of four types of shift work. Furthermore, for each year, we calculated the frequency of (i) any shifts (afternoon and/or night shifts), (ii) night shifts, and (iii) ≥3 consecutive night shifts. The cut-off points in categorizing exposure for shift work patterns were based on 1–25^th^, 25–50^th^, 50–75^th^ and >75^th^ percentile in the distribution of the exposed group. We also classified the cumulative number of years with any shift work and with any night work divided into three categories based on the exposure (never, 1–3, 4–7, ≥8 years), and sensitivity analyses of the cumulative number of years with exclusively night shifts (never, 1–3 and ≥4 years).

**Table 2:** Discrete-time proportional hazard models for incident type 2 diabetes (ICD: E11) among healthcare employees during follow-up 2013-2017, contributing to a total of 92 792 person-years (PY) for analyses of shift work patterns. Each of the models was estimated separately. A one-year time window was applied for the shift work patterns. [HR=hazard ratio; CI=confidence interval.]

Exposure ^a^		Type 2 diabetes			
		PY	N cases	HR ^b^ (95% CI)	HR ^c^ (95% CI)
**Type of shift work**					
	Always day ^d^ work	28 128	62	Ref.	Ref.
	Day and afternoon ^e^ shifts (no night shifts)	36 968	102	1.65 (1.21–2.28)	1.34 (0.97–1.88)
	Day and/or afternoon shifts, and night ^f^ shifts	20 362	33	1.24 (0.80–1.89)	1.13 (0.73–1.73)
	Night shifts only	7334	35	1.97 (1.28–2.98)	1.59 (1.02–2.43)
**Frequency of any shifts (afternoon and/or night)**					
	Always day work	28 128	62	Ref.	Ref.
	1–19 times/year	8307	22	1.73 (1.04–2.78)	1.56 (0.93–2.50)
	20–62 times/year	19 587	33	1.10 (0.71–1.67)	0.96 (0.62–1.46)
	63–120 times/year	26 713	71	1.71 (1.21–2.42)	1.37 (0.96–1.96)
	>120 times/year	10 057	44	2.06 (1.39–3.05)	1.67 (1.11–2.48)
	Trend test (risk increase per 10 times) ^g^			1.01 (1.00–1.02)	1.01 (0.99–1.02)
**Frequency of night shifts**					
	Never night shifts ^h^	65 096	164	Ref.	Ref.
	1–19 times/year	7737	14	1.15 (0.63–1.92)	1.20 (0.66–2.00)
	20–62 times/year	7607	11	0.88 (0.45–1.55)	0.92 (0.47–1.61)
	63–120 times/year	5528	12	0.90 (0.47–1.54)	0.87 (0.46–1.50)
	>120 times/year	6824	31	1.45 (0.97–2.10)	1.30 (0.86–1.89)
	Trend test (risk increase per 10 times) ^i^			1.02 (0.98–1.07)	1.01 (0.97–1.06)
**Frequency of ≥3 consecutive night shifts**					
	Never night shifts ^h^	65 096	164	Ref.	Ref.
	0 times/year	6289	8	0.67 (0.30–1.28)	0.71 (0.32–1.35)
	1–5 times/year	6914	12	1.10 (0.58–1.90)	1.12 (0.59–1.93)
	6–14 times/year	5181	11	1.11 (0.57–1.96)	1.10 (0.56–1.93)
	15–20 times/year	4096	16	1.43 (0.82–2.32)	1.34 (0.77–2.17)
	>20 times/year	5216	21	1.35 (0.83–2.08)	1.21 (0.74–1.87)
	Trend test (risk increase per 10 times) ^i^			1.10 (0.90–1.34)	1.05 (0.85–1.27)

^a^ Based on the exposure during the year preceding the outcome, except for in analyses of cumulative years of night work. The cut-off points in categorizing exposure for shift work patterns were based on 1–25^th^ percentile, 25^th^–50^th^ percentile, 50^th^–75^th^ percentile and >75^th^ percentile in the distribution of the exposed group, except for type of shift work. For analyses of cumulative number of years with any night work three categories were used based on the exposure. ^b^ Adjusted for calendar year (inherent in the analytical model), sex and age (continuous). ^c^ Additionally adjusted for country of birth (Sweden; Nordic countries except Sweden; Europe except Nordic countries; other countries) and profession (Nurses including midwives; Nursing assistants). ^d^ Day shifts: starts after 06:00 and ends no later than 18:00. ^e^ Afternoon shifts: starts after 12:00 and ends later than 18:00, but not a night shift. ^f^ Night shifts: at least three hours within 22:00 hours–06:00 hours. ^g^ Trend among those who worked any shifts. ^h^ Those who worked day and/or afternoon shifts, but no night shifts. ^i^ Trend among those who worked night shifts.

The method for aggregation and characterization of working time patterns has been evaluated (17) and was applied in previous studies on healthcare employees in Region Stockholm (16, 18, 19).

### Classification of the outcome

We identified first time diagnosis of T2D (E11 in ICD-10) and hypertension (I10 in ICD-10) by record linking to the National Patient Register at the National Board of Health and Welfare (inpatient and specialized outpatient care) and the Regional Database (VAL database) in Stockholm (non-hospitalized patients from inpatient and outpatient contacts). We included the diagnosis of T2D or hypertension that first appeared in either of the two registers, during the follow-up period 1 January 2013 to 31 December 2017. Prescribed medication was not used as an outcome.

### Data analysis

We used discrete-time proportional hazard models to calculate the risk of T2D and hypertension in relation to night and shift work patterns and duration of night work. The person-time was stratified by follow-up year and age and exposure variables were treated as time-dependent variables updated for each year. The employees were at risk from at least one year after employment, until the date of diagnosis, death, or end-of-follow-up, whichever came first. Employees who had been prescribed medication for the disease under study in the year before diagnosis were censored in the analysis.

In the estimation of hazard ratios (HR) with 95% confidence intervals (CI), two levels of adjustments were made. The first adjusted for calendar year (inherent in the analytical model), sex and age (continuous); the second additionally adjusted for country of birth (Sweden; Nordic countries except Sweden; Europe except Nordic countries; other countries) and profession (nurses including midwives; nursing assistants). Country of birth was used as a proxy measure for ethnicity, and profession as a proxy for socioeconomic status.

In the main analyses, we used a one-year time window where the estimated HR were based on the exposure/shift work patterns during the year preceding the outcome (also applied for the comparison group), except for analyses of cumulative exposure. Based on the main analyses, we also performed additional analyses focusing on a three-year time window (for frequencies: average value for the years with information on work hours, up to three years preceding the outcome) to explore the risk related to exposure patterns in a somewhat longer time perspective. We also performed sensitivity analyses focusing on cumulative number of years with exclusively night work.

In addition, we performed trend tests, using continuous variables in the regression model. In sensitivity analyses we performed trend tests based on those with exclusively night work.

SAS software, version 9.4 for Windows (SAS Institute Inc, Cary, NC, USA) was used for all statistical analyses.

## Results

The characteristics of the study participants are presented in table 1 for the T2D cohort and in supplementary table S1 for the hypertension cohort. In the T2D cohort, there were 16 633 employees who, at the end of follow-up, had never worked night shifts and 11 848 who had ever worked night shifts. The hypertension cohort comprised 13 134 employees who never worked night shifts and 10 146 who ever worked night shifts.

**Table 1:** Baseline characteristics of the study participants (N=28 481) in the cohort for type 2 diabetes (ICD: E11).

Variables		Never night work ^a^			Ever night work ^a^			Total cohort	
		N	%		N	%		N	%
Sex									
	Women	15 009	90.2		10 056	84.9		25 065	88.0
	Men	1624	9.8		1792	15.1		3416	12.0
Age (years) ^b^									
	<40	5028	30.2		5524	46.6		10 552	37.0
	40–49	4196	25.2		3112	26.3		7308	25.7
	≥50	7409	44.5		3212	27.1		10 621	37.3
Country of birth									
	Sweden	13 284	79.9		9168	77.4		22 452	78.8
	Nordic counties except Sweden	993	6.0		646	5.5		1639	5.8
	Europe except Nordic countries	739	4.4		668	5.6		1407	4.9
	Outside Europe	1617	9.7		1364	11.5		2 981	10.5
Profession									
	Nurses	9175	55.2		7206	60.8		16 381	57.5
	Nursing assistants	7458	44.8		4642	39.2		12 100	42.5

^a^ At the end of follow-up, based on all years with information on work hours 2008–2016. ^b^ At the beginning of follow-up.

In both cohorts, the percentage of men was somewhat higher among those who ever worked night than among those who never worked night. Employees who had ever worked night tended to be younger than those who had not. Having worked nights was more common among nurses than nursing assistants and employees with a country of birth outside the Nordic countries.

During the follow-up period 2013–2017 (mean 3.7 years of follow-up for the diabetes cohort and 3.6 years for the hypertension cohort), we identified 232 incident cases of T2D and 875 incident cases of hypertension, when applying a one-year time window in the analyses. Correspondingly we identified 370 cases of T2D and 1229 of hypertension in analyses of risks related to cumulative number of years with shift or night work. The mean number of years with employment/exposure information during the period 2008–2016 was 6.0 years for the diabetes cohort and 5.8 years for the hypertension cohort.

### Type of shift work and risk of diabetes and hypertension

Healthcare employees who worked night shifts only had an increased risk for T2D the following year compared to those with always day shifts (fully adjusted model: HR 1.59, 95% CI 1.02–2.43) (table 2). Employees who worked day and afternoon shifts, but no night shifts, had an increased risk for T2D the following year compared to those with only day shifts when adjusted for calendar year, age and sex. After additional adjustment for country of birth and profession the increased risk was no longer statistically significant (HR 1.34, 95% CI 0.97–1.88). For employees who worked a mix of day and/or afternoon and night shifts, the risk was lower and not statistically significant. Additional analyses applying a three-year time window for the exposure showed similar results although the increased risk for T2D among employees who only worked night shifts was no longer statistically significant in the fully adjusted model (supplementary table S2). For hypertension, no increased risk was observed in relation to the different types of shift work (table 3 and supplementary table S3).

**Table 3:** Discrete-time proportional hazard models for incident hypertension (ICD: I10) among healthcare employees during follow-up 2013–2017, contributing to a total of 73 679 person-years (PY) for analyses of shift work patterns. Each of the models was estimated separately. A one-year time window was applied for the shift work patterns. [HR=hazard ratio; CI=confidence interval.]

Exposure ^a^		Hypertension			
		PY	No cases	HR ^b^ (95% CI)	HR ^c^ (95% CI)
**Type of shift work**					
	Always day ^d^ work	20 923	302	Ref.	Ref.
	Day and afternoon ^e^ shifts (no night shifts)	29 840	340	1.08 (0.92–1.26)	1.04 (0.88–1.22)
	Day and/or afternoon shifts, and night ^f^ shifts	17 261	148	1.01 (0.82–1.23)	0.99 (0.81–1.21)
	Night shifts only	5655	85	1.01 (0.79–1.28)	0.96 (0.75–1.23)
**Frequency of any shifts (afternoon and/or night shifts)**					
	Always day work	20 923	302	Ref.	Ref.
	1–19 times/year	6705	78	1.19 (0.92–1.52)	1.15 (0.89–1.47)
	20–62 times/year	16 060	166	1.04 (0.86–1.26)	1.01 (0.84–1.23)
	63–120 times/year	22 087	225	1.04 (0.87–1.23)	0.99 (0.83–1.19)
	>120 times/year	7904	104	1.01 (0.80–1.26)	0.95 (0.75–1.19)
	Trend test (risk increase per 10 times) ^g^			1.00 (0.99–1.01)	1.00 (0.99–1.01)
**Frequency of night shifts**					
	Never night shifts ^h^	50 763	642	Ref.	Ref.
	1–19 times/year	6640	57	1.07 (0.80–1.39)	1.08 (0.81–1.40)
	20–62 times/year	6553	44	0.82 (0.59–1.10)	0.82 (0.60–1.11)
	63–120 times/year	4462	53	0.99 (0.74–1.29)	0.98 (0.73–1.28)
	>120 times/year	5261	79	0.99 (0.78–1.25)	0.96 (0.76–1.21)
	Trend test (risk increase per 10 times) ^i^			1.00 (0.98–1.03)	1.00 (0.98–1.02)
**Frequency of ≥3 consecutive night shifts**					
	Never night shifts ^h^	50 763	642	Ref.	Ref.
	0 times/year	5242	53	1.05 (0.79–1.38)	1.06 (0.79–1.39)
	1–5 times/year	5968	43	0.90 (0.65–1.21)	0.91 (0.66–1.22)
	6–14 times/year	4390	37	0.90 (0.64–1.24)	0.90 (0.63–1.24)
	15–20 times/year	3245	41	0.95 (0.68–1.28)	0.93 (0.67–1.26)
	>20 times/year	4071	59	1.01 (0.77–1.31)	0.99 (0.75–1.28)
	Trend test (risk increase per 10 times) ^i^			1.03 (0.92–1.15)	1.01 (0.91–1.13)

^a^ Based on the exposure during the year preceding the outcome, except for in analyses of cumulative years of night work. The cut-off points in categorizing exposure for shift work patterns were based on 1–25^th^ percentile, 25^th^–50^th^ percentile, 50^th^–75^th^ percentile and >75^th^ percentile in the distribution of the exposed group, except for type of shift work. For analyses of cumulative number of years with any night work three categories were used based on the exposure. ^b^ Adjusted for calendar year (inherent in the analytical model), sex and age (continuous). ^c^ Additionally adjusted for country of birth (Sweden; Nordic countries except Sweden; Europe except Nordic countries; other countries) and profession (Nurses including midwives; Nursing assistants). ^d^ Day shifts: starts after 06:00 and ends no later than 18:00. ^e^ Afternoon shifts: starts after 12:00 and ends later than 18:00, but not a night shift. ^f^ Night shifts: at least three hours within 22:00 hours – 06:00 hours. ^g^ Trend among those who worked any shifts. ^h^ Those who worked day and/or afternoon shifts, but no night shifts. ^i^ Trend among those who worked night shifts.

### Frequency of shift work and consecutive night shifts and risk of diabetes and hypertension

For employees with the highest frequency of any shifts (>120 times per year) there was a statistically significant risk increase for T2D the following year in the fully adjusted model (HR 1.67, 95% CI 1.11–2.48) compared to employees with always day work (table 2). The employees who had the highest frequency of night shifts (>120 times per year) had an increased risk for T2D the following year compared to those who never worked night shifts, although statistically not significant (fully adjusted model: HR 1.30, 95% CI 0.86–1.89) (table 2). However, the trend test indicated no increasing risk for diabetes with increasing number of any shifts or with increasing number of night shifts, expressed as risk increase per 10 shifts. The findings were similar when applying a three-year time window (supplementary table S2).

The risk of T2D tended to be higher the following year for those who frequently worked ≥3 consecutive night shifts (15–20 times per year and >20 times per year) compared to those who never worked nights, but no statistically significant risk increase was observed and no significant trend (table 2). In the analyses with a three-year time window, we observed an increased risk of T2D among employees who worked ≥3 consecutive night shifts on average 15–20 times per year (HR 1.71, 95% CI 1.05–2.63 in the fully adjusted model) (supplementary table S2).

For hypertension, no increased risk was observed in relation to the frequency of any shifts, night shifts or ≥3 consecutive night shifts (table 3 and supplementary table S3).

### Cumulative number of years with shift work or night work

We did not observe any increased risk for either T2D or hypertension in relation to the number of years with any shift work compared to those who always worked day or the number of years with any night work compared to those who never worked nights (table 4 and 5). However, in sensitivity analyses focusing on employees with exclusively night work, we observed a tendency of an increased risk of T2D, but not hypertension, among employees with a long duration of exclusively night work. Among employees with ≥4 years with exclusively night work, the HR for T2D was 1.36 (95% CI 0.91–1.95) in the fully adjusted model (table 4). The corresponding HR for hypertension in employees with ≥4 years with exclusively night work was 0.94 (95% CI 0.71–1.22) in the fully adjusted model (table 5).

**Table 4:** Discrete-time proportional hazard models for incident type 2 diabetes (ICD: E11) among healthcare employees during follow-up 2013–2017, contributing to a total of 131 434 person-years (PY) for analyses of number of years with any shift work, any night work, or exclusively night work. [HR=hazard ratio; CI=confidence interval.]

Exposure ^a^		Type 2 diabetes			
		PY	N cases	HR ^b^ (95% CI)	HR ^c^ (95% CI)
**Cumulative number of years with any shift work**					
	Always day work	29 210	105	Ref.	Ref.
	1–3 years	36 106	77	1.16 (0.86–1.56)	1.00 (0.74–1.35)
	4–7 years	56 808	152	1.15 (0.89–1.49)	0.95 (0.74–1.24)
	≥ 8 years	9310	36	1.25 (0.82–1.86)	0.98 (0.64–1.47)
	Trend test (risk increase per year) ^d^			1.02 (0.99–1.06)	0.99 (0.96–1.03)
**Cumulative number of years with any night work**					
	Never night shifts ^e^	79 953	251	Ref.	Ref.
	1–3 years	28 381	45	0.88 (0.63–1.20)	0.87 (0.63–1.19)
	4–7 years	20 369	62	1.09 (0.82–1.43)	1.07 (0.80–1.41)
	≥ 8 years	2731	12	1.08 (0.56–1.88)	1.03 (0.54–1.80)
	Trend test (risk increase per year) ^f^			1.01 (0.93–1.10)	1.01 (0.93–1.09)
**Cumulative number of years with exclusively night work**					
	Never night shifts ^e^	79 953	251	Ref.	Ref.
	Night but not exclusive any year	36 608	54	0.81 (0.60–1.08)	0.85 (0.62–1.13)
	1–3 years mix of exclusive and not exclusive	6690	18	1.04 (0.62–1.63)	1.03 (0.62–1.62)
	≥4 years mix of exclusive and not exclusive	1963	6	0.81 (0.32–1.66)	0.75 (0.30–1.55)
	1–3 years exclusively nights	2088	10	1.25 (0.62–2.24)	1.11 (0.55–1.98)
	≥4 years exclusively nights	4132	31	1.53 (1.03–2.20)	1.36 (0.91–1.95)
	Trend test (risk increase per year exclusively nights) ^g^			1.04 (0.93–1.15)	1.02 (0.92–1.14)

^a^ For analyses of cumulative number of years with any shift work or any night work three categories were used based on the exposure. ^b^ Adjusted for calendar year (inherent in the analytical model), sex and age (continuous). ^c^ Additionally adjusted for country of birth (Sweden; Nordic countries except Sweden; Europe except Nordic countries; other countries) and profession (Nurses including midwives; Nursing assistants). ^d^ Trend among those who worked any shifts. ^e^ Those who worked day and/or afternoon shifts, but no night shifts. ^f^ Trend among those who worked night shifts. ^g^ Trend among those who worked exclusively night shifts.

**Table 5:** Discrete-time proportional hazard models for incident hypertension (ICD: I10) in healthcare employees during follow-up 2013–2017, contributing to a total of 101 949 person-years (PY) for analyses of number of years with any shift work, any night work, or exclusively night work. [HR=hazard ratio; CI=confidence interval.]

Exposure ^a^		Hypertension			
		PY	No cases	HR ^b^ (95% CI)	HR ^c^ (95% CI)
**Cumulative number of years with any shift work**					
	Always day work	20 147	339	Ref.	Ref.
	1–3 years	29 634	260	1.05 (0.89–1.24)	1.02 (0.86–1.20)
	4–7 years	45 216	514	1.05 (0.91–1.21)	1.01 (0.88–1.17)
	≥ 8 years	6952	116	1.20 (0.95–1.50)	1.14 (0.90–1.43)
	Trend test (risk increase per year) ^d^			1.01 (0.99–1.04)	1.01 (0.99–1.03)
**Cumulative number of years with any night work**					
	Never night shifts ^e^	59 665	812	Ref.	Ref.
	1–3 years	23 963	189	0.98 (0.83–1.15)	0.99 (0.84–1.16)
	4–7 years	16 310	193	0.97 (0.82–1.13)	0.97 (0.82–1.13)
	≥ 8 years	2011	35	1.02 (0.71–1.42)	1.00 (0.69–1.39)
	Trend test (risk increase per year) ^f^			1.01 (0.97–1.06)	1.01 (0.96–1.05)
**Cumulative number of years with exclusively night work**					
	Never night shifts ^e^	59 665	812	Ref.	Ref.
	Night but not exclusive any year	30 995	266	1.02 (0.88–1.17)	1.03 (0.90–1.19)
	1–3 years mix of exclusive and not exclusive	5310	50	0.83 (0.61–1.09)	0.83 (0.61–1.09)
	≥ 4 years mix of exclusive and not exclusive	1453	24	1.04 (0.68–1.53)	1.03 (0.67–1.52)
	1–3 years exclusively nights	1548	19	0.87 (0.53–1.33)	0.85 (0.52–1.31)
	≥ 4 years exclusively nights	2978	58	0.99 (0.75–1.28)	0.94 (0.71–1.22)
	Trend test (risk increase per year exclusively nights) ^g^			1.02 (0.96–1.10)	1.02 (0.95–1.09)

^a^ For analyses of cumulative number of years with any shift work or any night work three categories were used based on the exposure. ^b^ Adjusted for calendar year (inherent in the analytical model), sex and age (continuous). ^c^ Additionally adjusted for country of birth (Sweden; Nordic countries except Sweden; Europe except Nordic countries; other countries) and profession (Nurses including midwives; Nursing assistants). ^d^ Trend among those who worked any shifts. ^e^ Those who worked day and/or afternoon shifts, but no night shifts. ^f^ Trend among those who worked night shifts. ^g^ Trend among those who worked exclusively night shifts.

## Discussion

The main findings of this study were an increased risk of T2D among healthcare employees who in the previous year worked permanent night shifts and those with a high frequency (>120 times per year) of any shifts (afternoon and/or night shifts), compared to those with always day work. We also observed a non-significant tendency of increased risk of T2D among employees with a mix of day and afternoon shifts, and among employees with the highest frequency of night shifts or frequent spells of ≥3 consecutive night shifts. The number of years with permanent night work tended to affect the risk of T2D. For the outcome hypertension, we observed no increased risk in association with intensive night and shift work or with number of years with any, or permanent, night work.

The findings for T2D indicated that both permanent night work and rotating shift work that does not include night work may affect the risk, even though the evidence was stronger for permanent night workers. Mixed shift work that includes nights seems to affect the risk mainly when the exposure is intense. The risk among permanent night workers may not just be explained by intensive night work, even though there is an overlap between the exposure groups, where permanent night workers are overrepresented in the highest exposure groups for the various shift work patterns. However, the employees with permanent night work also incorporate part-time workers, with a low-to-medium frequency of night shifts per year. Even so, the finding of a tendency of increased risk of T2D for those with the highest frequency of night shifts and most frequent spells of consecutive nights shifts indicates that intensive night work per se may at least partially contribute to this risk among permanent night workers.

Our results of an association between night shift work and T2D are in agreement with several previous studies (4–7, 20–23), although some of them also observed significant associations with shift work that did not include nights. However, one study from the UK, including findings for current permanent night shift work, did not observe any association for this subgroup. Instead, the frequency of night shifts per month seemed to be of most importance for the diabetes risk among employees in the UK (6). The UK study included employees in any occupation and about 50% were men. That differs from our cohort of mainly female healthcare employees, for example in terms of potential gender differences in relation to risk factors for cardiovascular disease and diabetes. Even so, in a cohort of mainly female healthcare workers in Finland, an increased use of medication for T2D was observed related to shift work that did not include nights, but only in the age group 40–49 years, and not for shift work that included nights (24).

Although we did not observe an association between shift work – with or without night shifts – and hypertension, several previous studies have (2, 3, 24, 25). Shift work both with and without night shifts was associated with an increased use of medication for hypertension in the Finnish cohort of mainly female healthcare workers among those aged <50 years (24). Among previous studies, some also indicated evidence for an association with permanent, or mostly, night work (3, 25), one of which was among Chinese female nurses (25). Ferguson et al's cohort study (3) among mostly male industrial workers applied a similar time window for the exposure (the last year) as we did and had access to objectively assessed information on shift work. They observed the highest risk of incident hypertension among workers with a combination of mostly night work and frequent rotations. In the cross-sectional study by Zhao et al (25) an increased risk of self-reported or self-measured hypertension was observed for female nurses at similar exposure levels as in our study where we did not observe any increase in risk. However, a review and meta-analysis by Gamboa Madeira et al (26) observed no significant increase in diagnosis of hypertension related to shift work with or without night work or permanent night work.

In the main analyses, we used a one-year time window for the exposure to assess the risk associated with recent exposure to various patterns of night and shift work. Working hour characteristics during the preceding year has previously been shown to affect the risk of incident hypertension (3) and cardiovascular disease (16, 19). Additionally, we applied a three-year time window to assess the risk related to work time patterns for a somewhat longer time perspective, which may possibly be more relevant to the risk of developing T2D. However, in both these scenarios, employees may have belonged to other similar exposure groups in previous years. Therefore, to some extent, the results reflect the risk related to a combination of more recent and past working time exposures. In this study, the effects of night and shift work patterns on diabetes risk were similar for work in the past year and the accumulated effects from the three previous years of exposure.

In the analyses of cumulative number of years with shift or night work, we did not observe any risk of diabetes or hypertension associated with number of years with any shift work or any night work. But we did see a tendency for an association between diabetes risk and number of years with exclusively night work. The reference group in the analyses of night work was those who worked day and/or afternoon shifts (never night shifts), and it is possible that the associations with cumulative night work would have been stronger if compared to those who worked day shifts only. However, we did not observe an association between cumulative number of years with any shift work compared to those with always day work. An association between duration of night shift work and T2D was previously observed in studies of American women (4, 20, 23), two of which were among nurses (4, 23).

Potential mechanisms for the association between T2D and night and shift work include disturbances in the circadian rhythm, that may lead to changes in the hormonal system, such as an increased production of cortisol and interleukins and a suppression of melatonin secretion at night, and thereby affect the glucose metabolism and insulin resistance (7, 15, 27). Both central and peripheral circadian clocks are involved in this regulation of the rhythm of hormone production (27). The timing and total production of hormones may vary with different night shift schedules and light conditions (28). Stress mechanisms and disturbed sleep may also be involved as well as behavioral changes related to night work. Long-term exposure to night work has been associated with higher body mass index and waist circumference (5).

In previous studies based on the same cohort we found that night and shift work affected the risk of cardiovascular disease (16, 19). In the present study, the risk of T2D, but not hypertension, was increased by night and shift work. Therefore, it is possible that T2D may have an important role in the pathway from night shift work to the increased risk of cardiovascular diseases noted in previous studies, while hypertension does not contribute as much to this association. It thus implies that the mechanism is mainly through a disturbance of metabolism rather than a disturbance in the regulation of blood pressure. However, the mechanisms are complex, and other studies found support for an association between night and shift work and hypertension.

In the present study, we excluded employees who were previously prescribed any kind of anti-diabetes or anti-hypertension medication. Many anti-hypertension medications may also be prescribed to treat other health issues, mainly cardiovascular diseases. Since we cannot be sure that the medicine was prescribed specifically to treat hypertension, there is a possibility that we selected a healthier cohort for hypertension than for diabetes, free from most previous cardiovascular disease. Therefore, it cannot be ruled out that we, to some extent, underestimated the actual risk of hypertension related to night and shift work.

This study had several strengths and limitations. The strengths of the study include the longitudinal nature and the access to very detailed information on working hours that we assessed and updated repeatedly. The exposure information came from employee registers and we did not need to use self-reported information on working hours, thus avoiding recall bias. Also, the diagnoses were based on a combination of national and regional registers, which covered both inpatient and outpatient visits. In addition, we had information on prescribed medicines, which increased the likelihood of including only incidental cases in the analyses. Since the cohort consists of nurses and nursing assistants, the percentage of study participants with night shift work was high and they represent a rather homogenous group with, probably, small differences in lifestyles and other potential individual and social risk factors. Even so, it is a limitation that we did not have any information on potential individual confounders, such as smoking, obesity, physical inactivity, or alcohol consumption. These may act as confounders or mediators. However, we did account for a previous history of diabetes or hypertension, and we adjusted for profession, which may reduce the risk of confounding related to lifestyle factors. A further limitation is the uneven gender distribution in the cohort. Employers in Sweden are required to offer medical checks for night workers and these usually include measurements of blood pressure and plasma glucose levels. This would potentially have led to a positive detection bias but also add to the healthy worker effect, as employees with health issues may have been transferred to day work or less intensive shift schedules. The healthy worker effect may have resulted in an underestimation of the risks related to night and shift work. There is also a possible underestimation of the risks related to cumulative number of years with night and shift work, related to a lack of exposure information before 2008.

### Concluding remarks

In this cohort of mainly female healthcare employees we observed an increased risk of T2D, but not hypertension, associated with permanent night work the previous year, intensive shift work with afternoon and/or night shifts the previous year, and a non-significant increase related to a high cumulative number of years with permanent night work. The risk of T2D was to some extent affected by frequent spells of several night shifts in a row.

Our findings are consistent with some previous observations from this cohort of healthcare employees, where intensive night work was associated with risk of preterm birth and cardiovascular disease. Work schedules that minimize intensive night shift work may possibly reduce those risks as well as of T2D. We recommend that employers provide regular medical checks for shift workers and offer preventive measures when justified. We welcome more studies with detailed and repeated information on working hours in a longitudinal approach, and preferably also for other professional groups and industries.

### Patient consent for publication

Not required.

### Ethical approval

Ethical approval was obtained from the Regional Ethics Committee in Stockholm (2016/2490-31/2; 2017/1157-32).

## Supplementary material

Supplementary material
