# Supplementary material for: Night and shift work patterns and incidence of type 2 diabetes and hypertension in a prospective cohort study of healthcare employees
Source: Scand J Work Environ Health. 2023 Aug 29;49(6):439–48. doi: 10.5271/sjweh.4104 (PMC10822121; doi:10.5271/sjweh.4104)
Supplement: Supplementary material [file SJWEH-49-439-S001.pdf]

Night and shift work patterns and incidence of type 2 diabetes and hypertension in a prospective cohort study of healthcare employees[1]

by Andreas Viklund, MD, Tomas Andersson, BSc, Jenny Selander, PhD, Manzur Kader, PhD, Maria Albin, MD, Theo Bodin, MD, Mikko Härmä, MD, Petter Ljungman, MD, Carolina Bigert, MD

1. Supplementary material
2. Correspondence to: Carolina Bigert, Institute of Environmental Medicine, Karolinska Institutet, Solnavägen 4 10th Floor, SE-113 65 Stockholm, Sweden. [E-mail: carolina.bigert@ki.se]

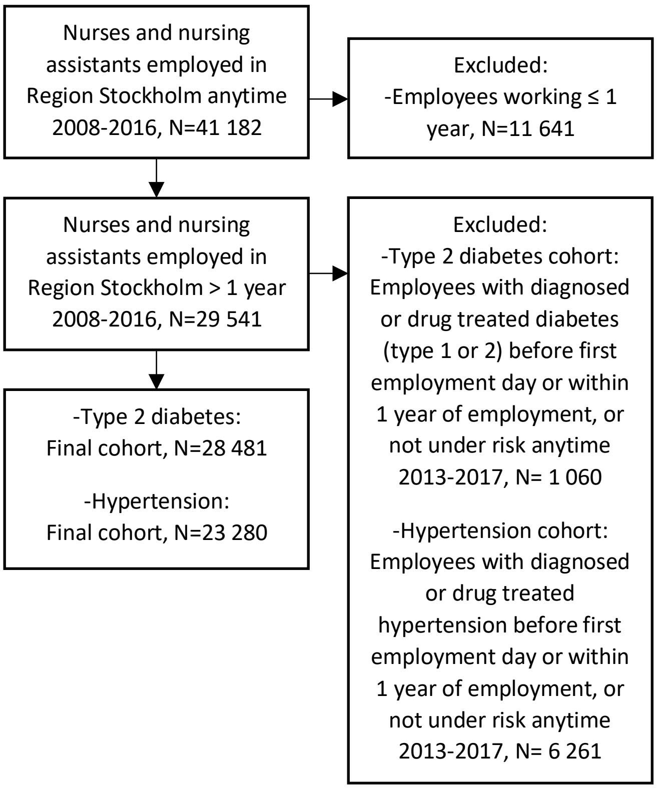

Figure S1. Flowchart for the inclusion and exclusion procedure, cohort for type 2 diabetes and cohort for hypertension

Table S1: Baseline characteristics of the study participants (N=23 280) in the cohort for hypertension (ICD: I10).

Variables	**Never night work[a]**		**Ever night work[a]**		**Total cohort**	
	N	%	N	%	N	%
**Sex**						
Women	11 799	89.8	8 575	84.5	20 374	87.5
Men	1 335	10.2	1 571	15.5	2 906	12.5
**Age[b]**						
<40	4 606	35.1	5 187	51.1	9 793	42.1
40-49	3 591	27.3	2 685	26.5	6 276	27.0
≥50	4 937	37.6	2 274	22.4	7 211	31.0
**Country of birth**						
Sweden	10 462	79.7	7 858	77.4	18 320	78.7
Nordic counties except Sweden	671	5.1	486	4.8	1 157	5.0
Europe except Nordic countries	615	4.7	582	5.7	1 197	5.1
Outside Europe	1 386	10.6	1 218	12.0	2 604	11.2
**Profession**						
Nurses	7 300	55.6	6 291	62.0	13 591	58.4
Nursing assistants	5 834	44.4	3 855	38.0	9 689	41.6

[a]At the end of follow-up, based on all years with information on work hours 2008-2016.
[b]At the beginning of follow-up.

Table S2: Discrete-time proportional hazard models for incident type 2 diabetes (ICD: E11) among healthcare employees during follow-up 2013-2017, contributing to a total of 92 792 person-years (PY) for analyses of shift work patterns. Each of the models was estimated separately. A three-year time window was applied.

			Type 2 diabetes	
Exposure[a]	PY	No of cases	HR[b] (95% CI)	HR[c] (95% CI)
**Type of shift work**				
Always day[d] work	24 821	59	Ref.	Ref.
Day and afternoon[e] shifts (no nights)	36 728	100	1.56 (1.13-2.18)	1.26 (0.91-1.78)
Day and/or afternoon shifts, and nights[f]	25 316	42	1.24 (0.83-1.85)	1.12 (0.74-1.67)
Night shifts only	5 927	31	1.96 (1.25-3.03)	1.55 (0.98-2.42)
**Frequency of any shifts (afternoon and/or night shifts), per year**				
Always day work	24 821	59	Ref.	Ref.
1-19 times	7 772	18	1.45 (0.83-2.41)	1.33 (0.76-2.22)
20-62 times	22 687	41	1.22 (0.81-1.82)	1.06 (0.70-1.58)
63-120 times	28 908	72	1.53 (1.08-2.18)	1.22 (0.85-1.75)
>120 times	8 604	42	2.09 (1.39-3.11)	1.66 (1.10-2.51)
Trend test (risk increase per 10 times)[g]			*1.02 (1.00-1.03)*	*1.01 (1.00-1.03)*
**Frequency of night shifts, per year**				
Never night shifts[h]	61 549	159	Ref.	Ref.
1-19 times	10 460	16	1.04 (0.60-1.70)	1.07 (0.61-1.75)
20-62 times	8 315	14	1.07 (0.59-1.79)	1.12 (0.62-1.87)
63-120 times	6 574	14	0.84 (0.46-1.40)	0.81 (0.45-1.36)
>120 times	5 894	29	1.48 (0.97-2.17)	1.32 (0.87-1.95)
Trend test (risk increase per 10 times)[i]			*1.02 (0.98-1.07)*	*1.01 (0.97-1.06)*
**Frequency of ≥3 consecutive night shifts, per year**				
Never night shifts[h]	61 549	159	Ref.	Ref.
0 times	6 450	8	0.66 (0.30-1.26)	0.69 (0.31-1.32)
1-5 times	9 536	15	1.11 (0.62-1.82)	1.15 (0.64-1.89)
6-14 times	6 190	12	0.99 (0.52-1.70)	0.96 (0.50-1.65)
15-20 times	4 151	21	1.82 (1.12-2.81)	1.70 (1.05-2.63)
>20 times	4 916	17	1.11 (0.64-1.78)	1.00 (0.58-1.60)
Trend test (risk increase per 10 times)[i]			*1.11 (0.91-1.36)*	*1.06 (0.86-1.29)*

[a]Based on the exposure during the years with information on work hours up to three years preceding the outcome (average value for frequencies). The cut-off points in categorizing exposure for shift work patterns were based on 1-25th percentile, 25th-50th percentile, 50th-75th percentile and >75th percentile in the distribution of the exposed group, except for type of shift work.

[b]Adjusted for calendar year (inherent in the analytical model), sex and age (continuous).

[c]Additionally adjusted for country of birth (Sweden; Nordic countries except Sweden; Europe except Nordic countries; other countries) and profession (Nurses including midwives; Nursing assistants).

[d]Day shifts: starts after 06:00 and ends no later than 18:00.

[e]Afternoon shifts: starts after 12:00 and ends later than 18:00, but not a night shift.

[f]Night shifts: at least three hours within 22:00 hours – 06:00 hours.

[g]Trend among those who worked any shifts.

[h]Those who worked day and/or afternoon shifts, but no night shifts.

[i]Trend among those who worked night shifts.

Table S3: Discrete-time proportional hazard models for incident hypertension (ICD: I10) among healthcare employees during follow-up 2013-2017, contributing to a total of 73 697 person-years (PY) for analyses of shift work patterns. Each of the models was estimated separately. A three-year time window was applied.

			Hypertension	
Exposure[a]	PY	No of cases	HR[b] (95% CI)	HR[c] (95% CI)
**Type of shift work**				
Always day[d] work	18 216	282	Ref.	Ref.
Day and afternoon[e] shifts (no nights)	29 512	330	1.03 (0.88-1.21)	0.98 (0.83-1.16)
Day and/or afternoon shifts, and nights[f]	21 447	190	1.03 (0.85-1.24)	1.01 (0.84-1.22)
Night shifts only	4 522	73	1.00 (0.77-1.29)	0.95 (0.72-1.23)
**Frequency of any shifts (afternoon and/or night shifts), per year**				
Always day work	18 208	282	Ref.	Ref.
1-19 times	6 281	63	0.97 (0.73-1.27)	0.95 (0.72-1.25)
20-62 times	18 703	187	1.05 (0.87-1.27)	1.02 (0.85-1.24)
63-120 times	23 824	245	1.02 (0.86-1.21)	0.98 (0.82-1.17)
>120 times	6 663	98	1.03 (0.82-1.30)	0.97 (0.76-1.23)
Trend test (risk increase per 10 times)[g]			*1.00 (0.99-1.01)*	*1.00 (0.99-1.01)*
**Frequency of night shifts, per year**				
Never night shifts[h]	47 728	612	Ref.	Ref.
1-19 times	9 068	75	1.08 (0.84-1.37)	1.10 (0.86-1.39)
20-62 times	7 170	52	0.94 (0.70-1.23)	0.95 (0.70-1.25)
63-120 times	5 229	66	1.02 (0.78-1.30)	1.01 (0.78-1.29)
>120 times	4 502	70	0.98 (0.76-1.24)	0.95 (0.73-1.21)
Trend test (risk increase per 10 times)[i]			*1.00 (0.97-1.02)*	*0.99 (0.97-1.01)*
**Frequency of ≥3 consecutive night shifts, per year**				
Never night shifts[h]	47 728	612	Ref.	Ref.
0 times	5 379	56	1.09 (0.82-1.42)	1.11 (0.83-1.44)
1-5 times	8 318	65	1.07 (0.82-1.37)	1.08 (0.83-1.39)
6-14 times	5 199	33	0.67 (0.46-0.94)	0.67 (0.46-0.93)
15-20 times	3 276	55	1.24 (0.93-1.62)	1.23 (0.92-1.60)
>20 times	3 797	54	0.97 (0.72-1.27)	0.94 (0.70-1.23)
Trend test (risk increase per 10 times)[i]			*0.99 (0.89-1.11)*	*0.98 (0.87-1.09)*

[a]Based on the exposure during the years with information on work hours up to three years preceding the outcome (average value for frequencies). The cut-off points in categorizing exposure for shift work patterns were based on 1-25th percentile, 25th-50th percentile, 50th-75th percentile and >75th percentile in the distribution of the exposed group, except for type of shift work.

[b]Adjusted for calendar year (inherent in the analytical model), sex and age (continuous).

[c]Additionally adjusted for country of birth (Sweden; Nordic countries except Sweden; Europe except Nordic countries; other countries) and profession (Nurses including midwives; Nursing assistants).

[d]Day shifts: starts after 06:00 and ends no later than 18:00.

[e]Afternoon shifts: starts after 12:00 and ends later than 18:00, but not a night shift.

[f]Night shifts: at least three hours within 22:00 hours – 06:00 hours.

[g]Trend among those who worked any shifts.

[h]Those who worked day and/or afternoon shifts, but no night shifts.

[i]Trend among those who worked night shifts.
